# Elevated plasma level of the glycolysis byproduct methylglyoxal on admission is an independent biomarker of mortality in ICU COVID-19 patients

**DOI:** 10.1038/s41598-022-12751-y

**Published:** 2022-06-09

**Authors:** Fadhel A. Alomar, Marai N. Alshakhs, Salah Abohelaika, Hassan M. Almarzouk, Mohammed Almualim, Amein K. Al-Ali, Fahad Al-Muhanna, Mohammed F. Alomar, Mousa J. Alhaddad, Mohammed S. Almulaify, Faisal S. Alessa, Ahmed S. Alsalman, Ahmed Alaswad, Sean R. Bidasee, Hassan A. Alsaad, Rudaynah A. Alali, Mona H. AlSheikh, Mohammed S. Akhtar, Mohammed Al Mohaini, Abdulkhaliq J. Alsalman, Hussain Alturaifi, Keshore R. Bidasee

**Affiliations:** 1 Department of Pharmacology and Toxicology, College of Clinical Pharmacy, Imam Abdulrahman Bin Faisal University, P. O. Box 1982, Dammam, 31441 Saudi Arabia; 2 Department of Internal Medicine, Dammam Medical Complex, Dammam, Saudi Arabia; 3 Clinical Pharmacology Department, Qatif Central Hospital, Ministry of Health, Qatif, Saudi Arabia; 4 Intenstive Care Unit, Qatif Central Hospital, Ministry of Health, Qatif, Saudi Arabia; 5 College of Medicine, Imam Abdulrahman Bin Faisal University, Dammam, Saudi Arabia; 6 Departments of Pharmacology and Experimental Neuroscience, University of Nebraska Medical Center, Omaha, NE USA; 7 Basic Sciences Department, College of Applied Medical Sciences, King Saud Bin Abdulaziz University for Health Sciences and King Abdullah International Medical Research Center, Al Ahsa, 31982 Saudi Arabia; 8 Department of Clinical Pharmacy, Faculty of Pharmacy, Northern Border University, Rafha, Saudi Arabia; 9 King Fahad Hospital Hofuf, Alahsa, Saudi Arabia

**Keywords:** SARS-CoV-2, Predictive markers, Mechanisms of disease

## Abstract

Biomarkers to identify ICU COVID-19 patients at high risk for mortality are urgently needed for therapeutic care and management. Here we found plasma levels of the glycolysis byproduct methylglyoxal (MG) were 4.4-fold higher in ICU patients upon admission that later died (n = 33), and 1.7-fold higher in ICU patients that survived (n = 32),compared to uninfected controls (n = 30). The increased MG in patients that died correlated inversely with the levels of the MG-degrading enzyme glyoxalase-1 (*r*^*2*^ = − 0.50), and its co-factor glutathione (*r*^*2*^ = − 0.63), and positively with monocytes (*r*^*2*^ = 0.29). The inflammation markers, SSAO (*r*^*2*^ = 0.52), TNF-α (*r*^*2*^ = 0.41), IL-1β (*r*^*2*^ = 0.25), CRP (*r*^*2*^ = 0.26) also correlated positively with MG. Logistic regression analysis provides evidence of a significant relationship between the elevated MG upon admission into ICU and death (P < 0.0001), with 42% of the death variability explained. From these data we conclude that elevated plasma MG on admission is a novel independent biomarker that predicts mortality in ICU COVID-19 patients.

## Introduction

The new coronavirus, termed severe acute respiratory syndrome coronavirus 2 (SARS-CoV-2), has claimed the lives of 6.2 million deaths worldwide. As of May 20th, 2022, more than 520 million individuals have been infected and 11.4 billion doses of vaccines have been administered^1^. Most individuals infected with SARS-CoV-2 remain asymptomatic or develop mild symptoms including fever, cough, muscle weakness, headache, sore throat, diarrhea, and loss of taste and smell. However, about 10% of individuals develop acute respiratory distress (ARDS) requiring intensive care unit (ICU) hospitalization^2–5^. This population includes those with advanced age, pre-existing medical conditions including cardiovascular diseases, cancers, diabetes mellitus and the unvaccinated^6–9^. Moreover, individuals with chronic diabetes mellitus (DM) are three times more likely to die from coronavirus disease 2019 (COVID-19) compared to infected individuals without DM^10–12^. In addition, many ICU patients that survived also develop post-COVID syndrome or long COVID whose symptoms include persistent cognitive impairment, immunosuppression, lung damage, heart, kidney, and vascular diseases^13,14^.

Because the number of COVID-19 patients requiring ICU treatment is extremely large (~ 10% of all infections) and can exceed the capacity of most hospitals, biomarkers are currently used to assist with stratifying and triage patients for management and care. Existing biomarkers fall into five broad categories: (1) hematological/coagulation markers including platelet to lymphocyte ratios, ferritin and D-dimer, (2) inflammation markers including serum lactate dehydrogenase (LDH), C-reactive protein (CRP), procalcitonin, interleukins (IL), IL-1b, IL-2, IL-6, IL-8, IL-10, IL-17, CXCL10/IP-10, and tumor necrosis factor (TNF-α), (3) cardiac dysfunction markers including troponin I (cTnI), N-terminal pro brain natriuretic peptide (NT-proBNP), α-hydroxybutyrate dehydrogenase (α-HBDH) and creatine kinase-myocardial band (CK-MB), (4) liver function markers including aspartate aminotransferase (AST), alanine aminotransferase (ALT), total bilirubin, gamma-glutamyl transferase (GGT), and serum albumin, and (5) renal function markers including glomerular filtration rate and blood urea nitrogen^15–17^. In a meta-analysis of thirty-two studies, Malik and colleagues found significant associations between blood lymphopenia, thrombocytopenia, elevated CRP, procalcitonin, lactate dehydrogenase, D-dimer, ALT, and AST with adverse COVID-19 outcomes. Only four of these thirty-two studies reported ICU death as an endpoint^18^. In another report, Narvel and colleagues indicated that although currently used biomarkers are useful for stratifying/triaging patients for ICU admittance, they are not specific for SARS-CoV-2^19^. Biomarkers associated with replication of SARS-CoV-2 and responses of the host cells to the infection could provide objective and unbiased information to health care professionals to better manage and predict outcomes of ICU COVID-19 patients. These markers may also provide insights into new strategies to attenuate the acute and long-term complications arising from SARS-CoV-2 infection.

SARS-CoV-2 infects human cells that express the angiotensin-converting enzyme 2 (ACE2) including pneumocytes, endothelial cells, and peripheral blood mononuclear cells (PBMCs)^20–22^. Like most viruses, after entry SARS-CoV-2 reprograms metabolism in the infected cells to obtain the building blocks needed for replication. These metabolic changes include upregulating glycolysis for faster production of ATP, upregulating the oxidative arm of the pentose phosphate pathway (PPP) for synthesis of nucleotides, amino acids, and lipids, and attenuating oxidative phosphorylation in the mitochondria of the infected host cells^23–25^. In addition to metabolic changes in the infected cells, the immune cells of the host also upregulate these pathways to orchestrate their highly specific series of responses to clear SARS-CoV-2 infection and repair the cellular damages^26–30^. On sensing pathogen-associated molecular patterns (PAMPs), the rapid response neutrophils move to the infected site (cells) to start the viral elimination process using multiple mechanisms including phagocytosis, oxidative burst, and the release of elastase and myeloperoxidase via azurophilic granules^31,32^. Polarized M1 pro-inflammatory macrophages rearrange their metabolism to utilize glycolysis rather than oxidative phosphorylation for rapid ATP synthesis, and the PPP for the oxidative burst needed for destroying the infectious agent^30^. The latter process is commonly termed the Warburg effect^28^. M1 macrophages also upregulate glycolysis by increasing hypoxia-inducible transcription factor 1α (HIF-1α) and glycolysis-related proteins in an O_2_-independent manner^28,33^. Danger-associated molecular patterns (DAMPs) from damaged and dying cells also activate Toll-like receptors and inflammasomes to increase inflammation and oxidative stress^34^.

In addition to ATP, anaerobic glycolysis also generates the highly cytotoxic byproduct, methylglyoxal (MG) from the interconversion of glyceraldehyde 3-phosphate and dihydroxyacetone phosphate by topoisomerase-1 (TPI-1)^35^. In healthy, uninfected individuals, plasma and tissue of MG levels are kept low by the actions of dual-enzyme glyoxalase system^36^. In the first step, the rate-limiting enzyme glyoxalase-I, (*GLOI,* EC4.4.1.5, Glo-I) converts a hemithioacetal formed between MG and glutathione (GSH) into S, D-lactoylglutathione. In the second step, S, D-lactoylglutathione is degraded by glyoxalase-II (*GLOII*, EC3.1.2.6, Glo-II) in the presence of H_2_O to D-lactic acid^37^. Reduced glutathione is synthesized in two steps. In the first step, glutamine and cysteine are converted into γ-glutamylcysteine by γ-glutamate cysteine ligase. In the second step, γ-glutamylcysteine and glycine are converted into glutathione by the enzyme glutathione synthase^38^. Expression of rate-limiting Glo1 is negatively regulated by the inflammation oxidative stress, and hypoxia^39–41^ conditions that are commonly seen in COVID-19 patients. The increase in oxidative stress will also increase demand for GSH. The increase in MG synthesis arising from upregulation of glycolysis coupled with decreases in free GSH and Glo1 levels should lead to accumulation of MG in blood and tissues of COVID-19 patients. At supraphysiologic levels, MG disrupts the function of endothelial and epithelial cells, resulting in microvascular leakage, and clots^42–45^. Supraphysiological levels of MG will also potentiate inflammation in many cell types by activating NF-κB^46^, the NLR family pyrin domain containing 3 (NRLP3) inflammasome^47^, and by inducing expression of the ectoenzyme vascular adhesion protein 1 and its cleaved analog semicarbazide-sensitive amine oxidase (SSAO)^48^. Elevated MG is also an underlying cause for tissue fibrosis^49^, a pathobiology reported in COVID-19 patients.

To this end, a cross-sectional study was conducted to (i) determine if MG levels are elevated in plasma of ICU COVID-19 patients upon admittance, and (ii) determine if elevation in plasma MG on admittance is predictive of subsequent death. Glutathione, Glo1, and immune cells were also measured to gain insight into mechanisms that contribute to MG accumulation. The inflammation markers SSAO, TNF-α, IL-1β and CRP were also measured to determine their relationship with plasma MG.

## Results

### Characterization of patients used in study

The general characteristics and medication history of patients used for this study are shown in Table 1. The sixty-five ICU patients were separated into those with diabetes mellitus (DM) and those without diabetes mellitus (non-DM) (Table 1). There were no significant differences in the mean age and weights of uninfected DM patients and infected patients. Uninfected individuals without DM were slightly younger and weighed less than ICU patients. There were twice as many males than females in this cohort of patients. A higher percentage of non-DM patients were on antiviral regimens than DM patients (41% vs 12%). Similar percentages of non-DM than DM patients were on antibiotics and steroids. Co-morbidities are also shown in Table 1. Of the thirty-four non-DM patients, sixteen died (47%), and seventeen of thirty-one DM patients died (55%).

**Table 1:** Demographic data of the uninfected controls and ICU COVID-19 patients.

	Control	DM	COVID-19	
	Uninfected (n = 30)	Uninfected (n = 24)	Non-DM (n = 34)	DM (n = 31)
**Sex**				
Male	22	17	18	21
Female	8	7	16	10
**Age (in years)**				
Median (range)	43 (27–73)	55 (33–80)	46 (30–80)	54 (28–81)
**Weight (kg)**				
Median (range)	70 (60–95)	80 (60–120)	80 (55–115)	80 (59–120)
**Treatments (%)**				
Antivirals Lopinavir/Ritonavir Ribavirin Interferon Favipiravir			41	12
Antibiotics Ceftriaxone Azithromycin Linezolid Vancomycin Tazocin Meropenem			85	80
Steroids Dexamethasone Methylprednisolone			96	84
**Comorbidities (%)**				
Respiratory disease		4	11	8
Hypertension		54	30	76
Ischemic heart disease		13	15	8
Hyperlipidemia		25	4	12
Renal failure			11	24
**Clinical outcomes**				
Discharged n (%)			18 (53%)	14 (45%)
Died n (%)			16 (47%)	17 (55%)

The blood analyte profile of patients used in this study are shown in Table 2. They were divided into five groups: individual tests, blood cell/coagulation parameters, renal function parameters, liver function parameters and lipid profiles. Blood glucose levels in ICU COVID-19 patients without DM were higher than that of un-infected individuals. Neutrophils were higher, and lymphocytes basophils and eosinophils were lower in ICU COVID-19 patients. There were no changes in monocytes levels between non-infected controls and ICU COVID-19 patients. Ferritin was also elevated, and hemoglobin and hematocrit were lower in ICU COVID-19 patients. There were no significant differences in Na^+^, K^+^, Cl^−^, Ca^2+^, phosphorous, and Mg^2+^ levels between uninfected and ICU COVID-19 patients. However, albumin levels were lower, and urea was higher. Liver enzymes were also higher in ICU COVID-19 patients.

**Table 2:** Analytes in blood of uninfected (control and DM) and ICU COVID-19 patients.

Analytes	Control	DM	COVID-19	
	Uninfected (n = 30)	Uninfected (n = 24)	Non-DM (n = 34)	DM (n = 31)
**Individual**				
Glucose random (3.3–9.99 mmol/L)	5.4 ± 1.2	13.6 ± 5.4↑	8.1 ± 2.7↑	14.5 ± 7.0↑
Lactate (0.5–2.2 mmol/L)			1.7 ± 0.6	2.2 ± 1.5
**Blood cell/coagulation**				
WBC (4–10 × 10^3^/μL)	5.1 ± 1.3	8.03 ± 1.9↑	9.2 ± 3.7↑	10.9 ± 4.6↑
RBC (4.5–5.5 × 10^6^/μL)	5.7 ± 0.8	4.6 ± 0.8	4.4 ± 0.9	4.4 ± 0.9
Platelet (150–430 × 10^3^/μL)	249.3 ± 64.5	284.7 ± 126.9↑	286.9 ± 113.3	274.5 ± 139.7
Neutrophils (1.5–6) × 10^9^/L	2.03 ± 0.17	4.8 ± 1.8	7.6 ± 1.3↑	7.59 ± 0.73↑
Lymphocytes (1.3–2.9) × 10^9^/L	2.06 ± 0.08	1.5 ± 0.62	1.16 ± 0.17↓	1.35 ± 0.14↓
Monocytes (0.1–0.6) × 10^9^/L	0.399 ± 0.12	0.38 ± 0.18	0.385 ± 0.04	0.46 ± 0.04
Eosinophils (0.02–0.5) × 10^9^/L	0.11 ± 0.015	0.05 ± 0.005	0.017 ± 0.01↓	0.033 ± 0.015↓
Basophils (0–0.2) × 10^9^/L	0.032 ± 0.003	0.02 ± 0.014	0.023 ± 0.01↓	0.017 ± 0.002↓
Ferritin (30–400 μg/L)		1238 ± 1224↑	1618 ± 1600↑	1216 ± 1083↑
Hemoglobin (13–17 g/dL)	13.9 ± 1.1	10.9 ± 1.7	11 ± 2.4↓	10.5 ± 1.8↓
Hematocrit (49–54%)	45.1 ± 3.2	37.9 ± 6.1	35.1 ± 7.4↓	33.7 ± 7.5↓
Prothrombin time (PT) (11.5–15.5 s)	14.8 ± 2.9	15 ± 3.8	15.4 ± 4.7	15.1 ± 3.4
Partial thromboplastin time (PTT) (26.4–36 s)	37.8 ± 4.4	31.9 ± 7.3	34.5 ± 7.1	31.9 ± 6.6
International normalize test (INR) (0.85–1.15)	1.1 ± 0.2	1.1 ± 0.4	1.2 ± 0.5	1.1 ± 0.3
**Renal function**				
Sodium (135–153 mmol/L)	141.1 ± 1.7	140.1 ± 6.2	39.9 ± 7.1	140.6 ± 6.9
Potassium (3.5–5.3 mmol/L)	4.3 ± 0.3	4.4 ± 0.5	4.2 ± 0.7	4.4 ± 0.5
Chloride (96–106 mEq/L)	100.9 ± 1.5	101.6 ± 5.9	102.6 ± 7.7	102.4 ± 6.7
Calcium (2.1–2.55 mmol/L)	2.4 ± 0.1	2.1 ± 0.1	2.1 ± 0.2	2.1 ± 0.2
Phosphorus (0.8–1.6 mmol/L)	1.1 ± 0.2	1.3 ± 0.5	1.2 ± 0.4	1.3 ± 0.5
Magnesium (0.7–1.0 mmol/L)		0.9 ± 0.11	0.9 ± 0.1	0.9 ± 0.1
Albumin (35–52 g/dL)	47.1 ± 2.4	30.1 ± 4.3	31.8 ± 5.2↓	30.3 ± 4.6↓
Urea (2.5–6.4 mmol/L)	4.7 ± 1.4	12.7 ± 7.7↑	9 ± 6.9↑	12.6 ± 6.4↑
Creatinine (53–106 μmol/L)	84.5 ± 19.8	137.9 ± 87.9↑	89.8 ± 58.8	131.5 ± 72.3↑
**Liver function**				
Total bilirubin (0–20 μmol/L)	12.1 ± 12.6	12.3 ± 10.6	22.8 ± 5.71↑	11.7 ± 9.3
Alanine aminotransferase (10–50 U/L)	23.5 ± 15.1	65.2 ± 130.1	41.6 ± 33.6	148.2 ± 446.8↑
Aspartate aminotransferase (0–38 U/L)	22.5 ± 14.2	42.6 ± 19.6	49.2 ± 39.9↑	316.2 ± 1331.5↑
Lactate dehydrogenase (81–230 U/L)		648.6 ± 108↑	611.4 ± 655.9↑	586 ± 772.2↑
Creatine phosphokinase (38–308 U/L)	160.5 ± 213.8	383.6 ± 427.7↑	346.3 ± 826.3↑	357.4 ± 393.9↑
Creatine phosphokinase-MB (7–25 U/L)	15.6 ± 3.9	30.8 ± 22.7↑	31.7 ± 30.2↑	33.6 ± 23.8↑
**Lipid profile**				
Cholesterol (1.3–5.2 mmol/L)		3.4 ± 1.3	4.2 ± 2.4	3.2 ± 1.0
Triglycerides (0.34–1.69 mmol/L)		1.63.2 ± 0.6	2.6 ± 1.5	1.9 ± 1.0
HDL-cholesterol (1.03–1.55 mmol/L)		0.82 ± 0.33	0.8 ± 0.3	0.8 ± 0.3
LDL-cholesterol (1.3–2.6 mmol/L)		1.83 ± 1.1	2.2 ± 1.1	1.7 ± 0.9

### Plasma levels of MG, glutathione, Glo1, SSAO, TNF-α, IL-1β, CRP in ICU COVID-19 patients

**Figure 1:**
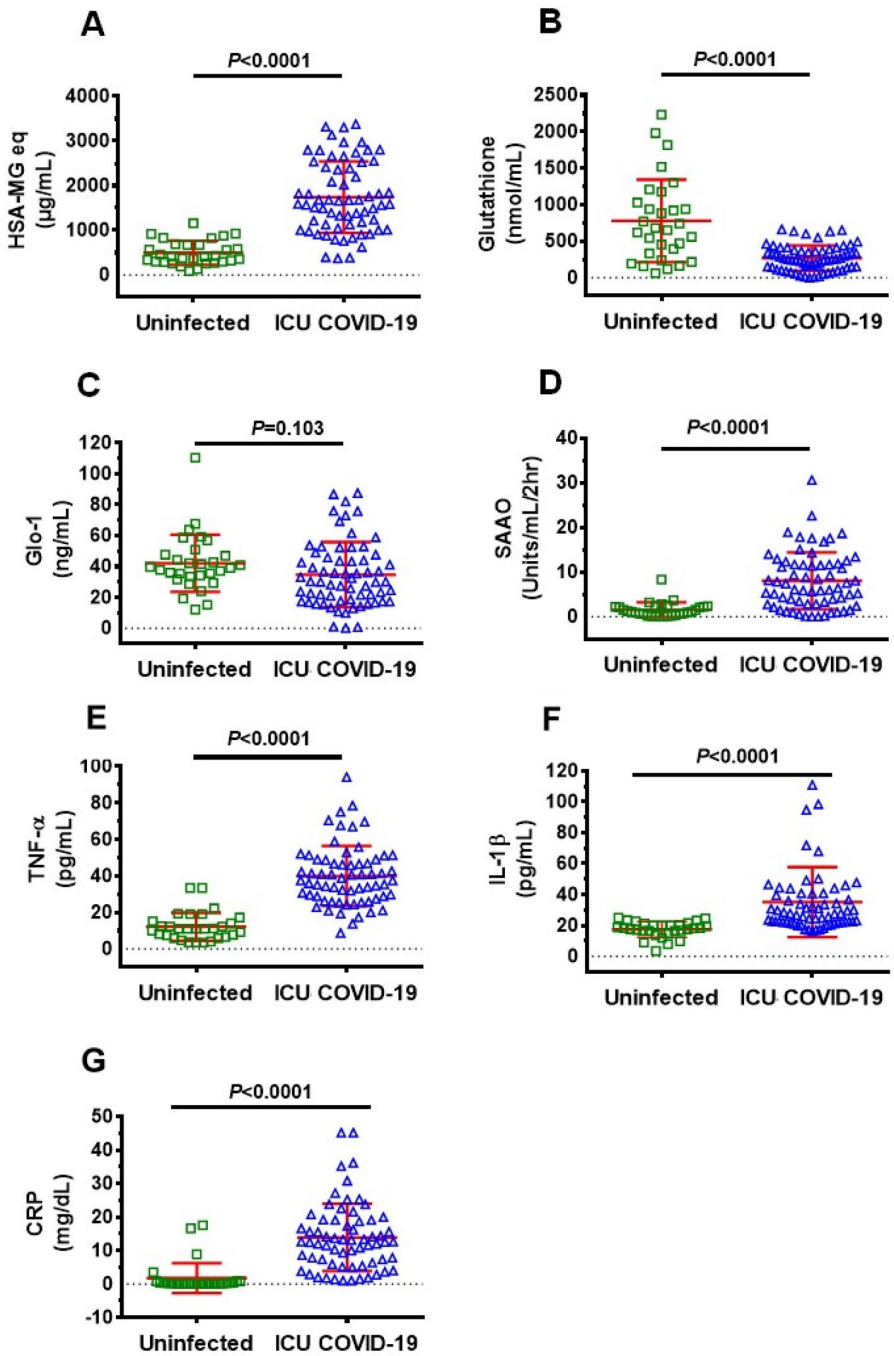
Plasma levels of MG, glutathione, Glo1 SSAO, TNF-α, IL-1β and CRP in uninfected control and ICU COVID-19 patients. (**A**) MG levels (detected as its surrogate, HSA-MG) were significantly higher in ICU COVID-19 patients compared to uninfected controls. (**B**) Glutathione levels were significantly lower in ICU COVID-19 patients compared to uninfected controls. (**C**) Glo1 levels in ICU COVID-19 patients were not significantly different from uninfected controls. (**D**–**G**) SSAO, TNF-α, IL-1β and CRP levels were significantly higher in ICU COVID-19 patients compared to uninfected controls, respectively. Data shown are for each patient with mean ± S.E.M from n = 30 uninfected controls (26.6% females) and n = 65 ICU COVID-19 patients (29.3% females). Statistical significances are shown above sets of data points on the graphs.

**Figure 2:**
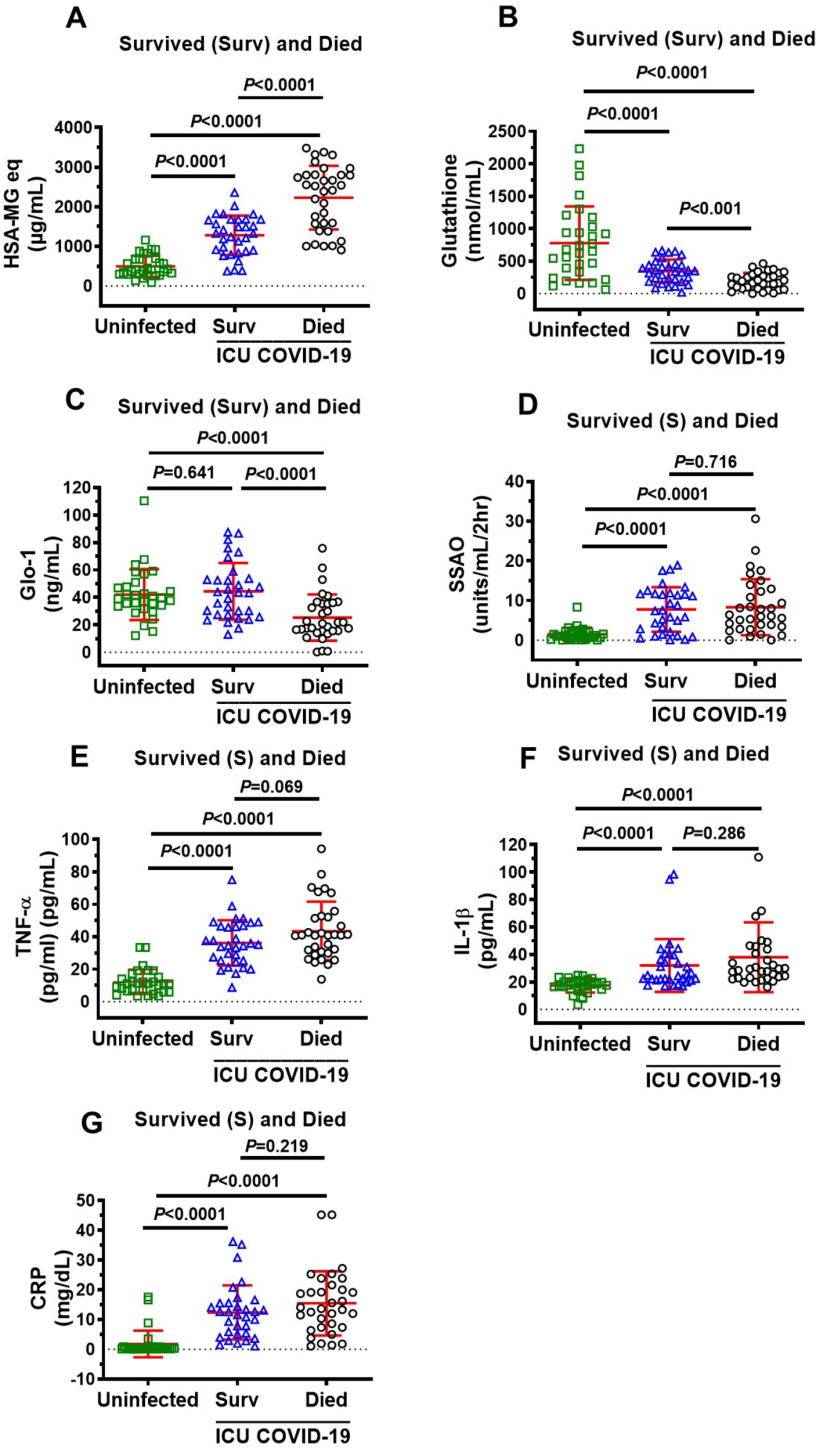
Plasma levels of MG, glutathione, Glo1, SSAO activity, TNF-α, IL-1β and CRP in uninfected controls and ICU COVID-19 patients that survived and died. (**A**) MG levels in plasma from ICU COVID-19 patients that survived and died were significantly higher than that in uninfected controls. MG levels in plasma from ICU COVID-19 that died were also significantly higher than ICU COVID-19 patients that survived. (**B**) Glutathione in plasma from ICU COVID-19 patients that survived and died were significantly lower than that in uninfected controls. Glutathione levels in plasma from ICU COVID-19 that died were also significantly lower than ICU COVID-19 patients that survived. (**C**) Glo1 in plasma from ICU COVID-19 patients that survived were not significantly different from that in uninfected controls. Glo1 levels in plasma from ICU COVID-19 patients that died were significantly lower than that in uninfected controls and ICU COVID-19 patients that survived. (**D**) SSAO activities in plasma from ICU COVID-19 patients that survived and died were significantly higher than that in uninfected controls. However, there were no significant difference in plasma SSAO activities between ICU COVID-19 patients that survived and died. (**E**) TNF-α levels in plasma from ICU COVID-19 patients that survived and died were significantly higher than that in uninfected controls. However, there were no significant difference in plasma levels of TNF-α in ICU COVID-19 patients that survived and died. (**F**) IL-1β levels in plasma from ICU COVID-19 patients that survived and died were significantly higher than that in uninfected controls. However, there were no significant difference in plasma levels of IL-1β in ICU patients that survived and died. (**G**) CRP levels in plasma from ICU COVID-19 patients that survived and died were significantly higher than that in uninfected controls. However, there were no significant difference in plasma levels of CRP in ICU patients that survived and died. Data shown are mean ± S.E.M from n = 30 in uninfected controls (26.6% females), n = 33 died (42.4% females) and n = 32 in survived (37.5% females). Statistical significance levels are shown above data points on each graph.

Plasma levels of MG, glutathione, and the inflammation markers SSAO, TNF-α, IL-1β, CRP, but not Glo1, were significantly higher in all ICU patients compared to uninfected individuals (Fig. 1A–G). MG was significantly higher (77%), and glutathione and Glo1 were significantly lower (44% and 43% respectively) in plasma in patients that died compared to ICU patients that survived (Fig. 2A–C). There were no significant differences in plasma levels of SSAO, TNF-α, IL-1β, and CRP between patients that died and survived (Fig. 2D–G).

### Immune cells in ICU COVID-19 patients

**Figure 3:**
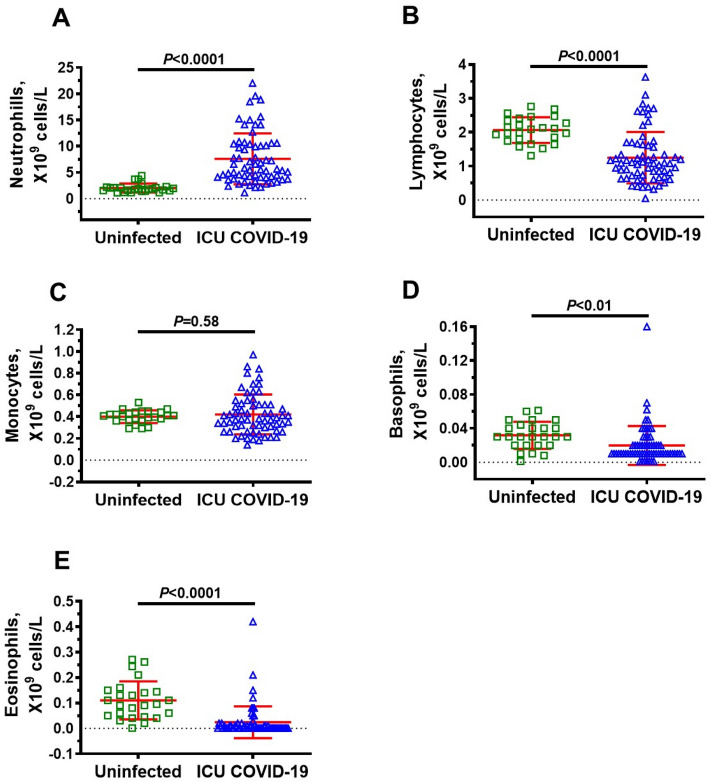
Plasma levels of neutrophils, lymphocytes, monocytes, basophils and eosinophils in uninfected control and ICU COVID-19 patients. (**A**) Neutrophils levels were significantly higher in ICU COVID-19 patients than that in uninfected controls. (**B**) Lymphocytes levels were significantly lower in ICU COVID-19 patients than that in uninfected controls. (**C**) Monocytes levels in ICU COVID-19 patients were not significantly different from uninfected controls. (**D**, **E**) Basophils and eosinophils levels were significantly lower in ICU COVID-19 patients compared to uninfected controls, respectively. Data shown are for each patient with mean ± S.E.M from n = 30 uninfected controls (26.6% females) and n = 65 ICU COVID-19 patients (29.3% females). Statistical significances are shown above sets of data points on the graphs.

**Figure 4:**
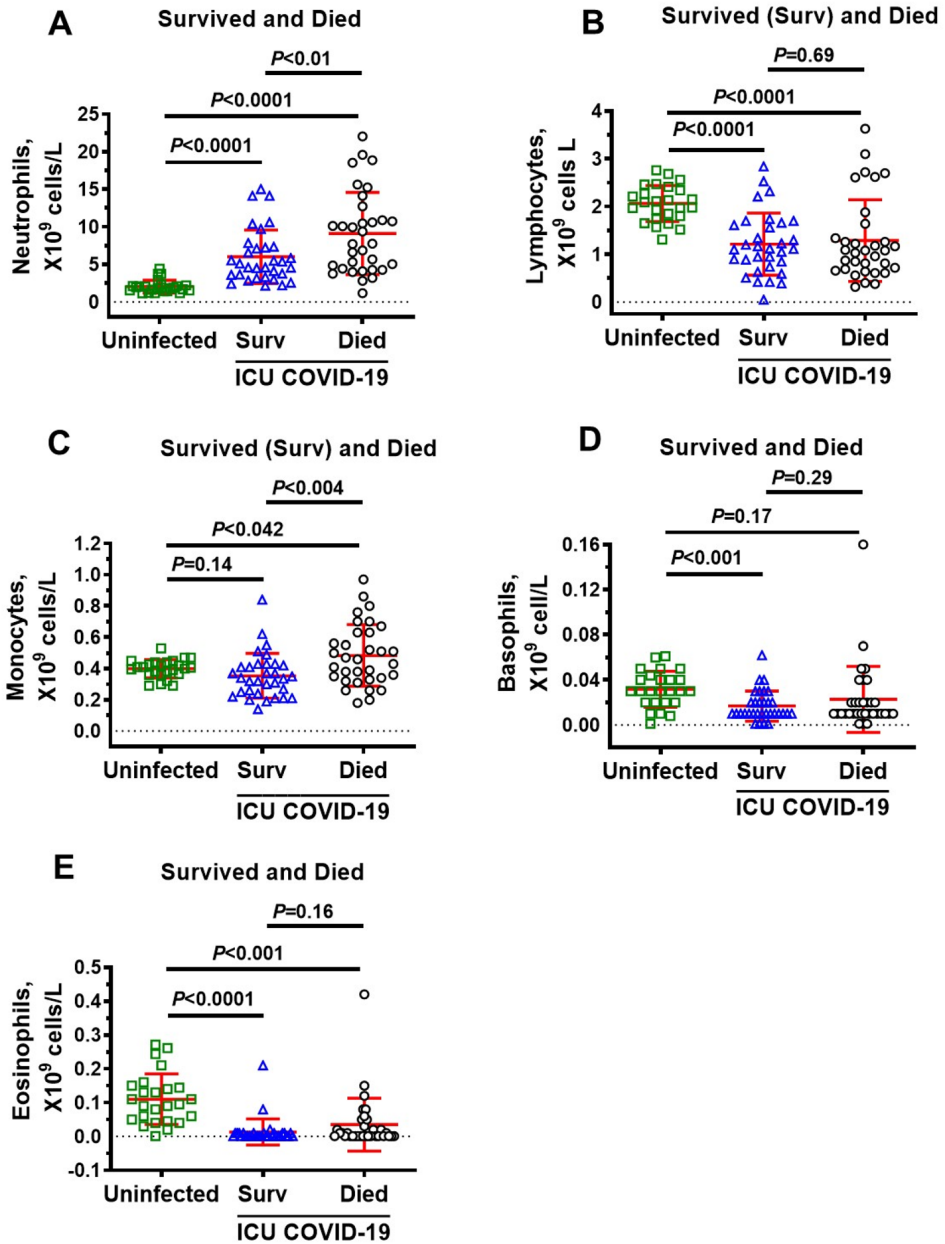
Plasma levels of neutrophils, lymphocytes, monocytes, basophils and eosinophils in uninfected non-DM individuals and ICU COVID-19 patients that survived and died. (**A**) Neutrophils levels in blood from ICU COVID-19 patients that survived and died were significantly higher than that in uninfected controls. Neutrophils levels in blood from ICU COVID-19 that died were also significantly higher than ICU COVID-19 patients that survived. (**B**) Lymphocytes in blood from ICU COVID-19 patients that survived and died were significantly lower than that in uninfected controls. However, there were no significant difference in blood levels of lymphocytes in ICU COVID-19 patients that survived and died. (**C**) Monocytes in blood from ICU COVID-19 patients that survived were not significantly different from that in uninfected controls. However, monocytes levels in blood from ICU COVID-19 patients that died were significantly higher than that in uninfected controls and ICU COVID-19 patients that survived. (**D**) Basophils levels in blood from ICU COVID-19 patients that survived but not died were significantly higher than that in uninfected controls. There was no significant difference in blood basophils levels between ICU COVID-19 patients that died and survived. (**E**) Eosinophils levels in blood from ICU COVID-19 patients that survived and died were significantly higher than that in uninfected controls. However, there were no significant difference in blood levels of eosinophils in ICU COVID-19 patients that survived and died. Data shown are mean ± S.E.M from n = 30 in uninfected controls (26.6% females), n = 33 died (42.4% females) and n = 32 in survived (37.5% females). Statistical significance levels are shown above data points on each graph.

Since upregulation of glycolysis in immune cells is required to orchestrate their highly specific series of responses to clear viral infection and repair cellular damage, we measured immune cells levels in ICU patients. Neutrophils were significantly higher, and lymphocytes, basophils, and eosinophils were significantly lower in ICU COVID-19 patients compared to uninfected individuals (Fig. 3A,B,D,E, respectively). No significant difference was found in the amounts of monocytes in ICU COVID-19 patients compared to uninfected controls (Fig. 3C). When ICU patients were separated into those that survived and died, there were no significant differences in the amounts of lymphocytes, basophils, and eosinophils (Fig. 4B,D,E). However, the amounts of neutrophils and monocytes were significantly higher in patients that died. (Fig. 4A,C).

### Plasma levels of MG, glutathione, Glo1, SSAO, TNF-α, IL-1β, CRP in non-DM, uninfected DM and ICU COVID-19 patients

**Figure 5:**
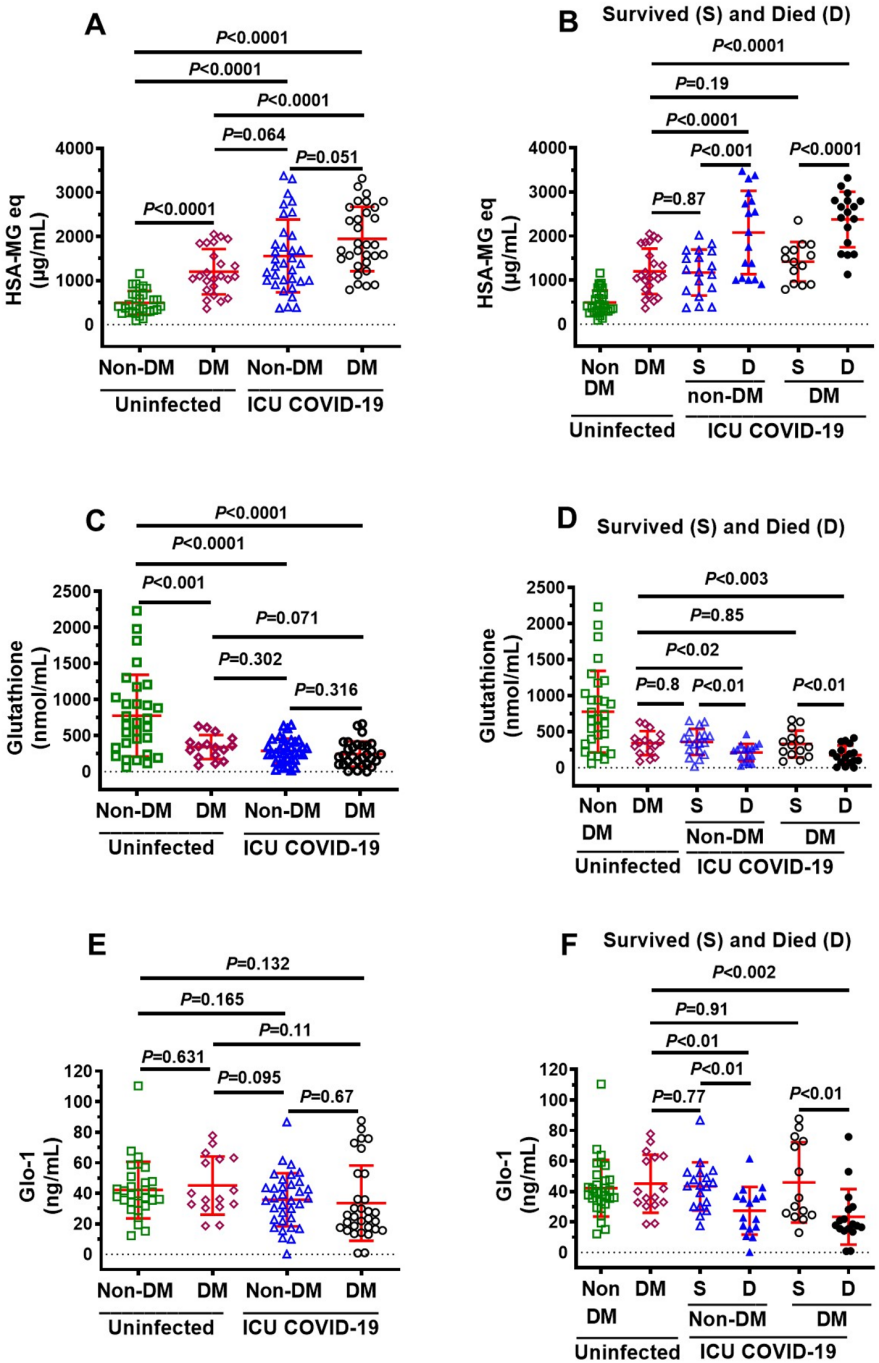
Plasma levels of MG, glutathione, and Glo-1 in ICU COVID-19 patients without diabetes mellitus (non-DM) and with diabetes (DM) and separated into those that survived and those that died. (**A**) MG levels were significantly higher in non-DM ICU COVID-19 patients compared to uninfected non-DM but not uninfected DM individuals. There was a significant difference in MG levels between DM ICU COVID-19 patients and uninfected non-DM or uninfected DM individuals. There was not a significant difference in MG levels between non-DM ICU COVID-19 and DM ICU COVID-19. (**B**) Plasma of MG in non-DM and DM ICU COVID-19 patients that died were significantly higher than that survived. There was not a significant difference in MG levels between DM ICU COVID-19 patients that survived and uninfected DM individuals. However, there was a significant difference in MG levels between non-DM ICU COVID-19 patients that died and uninfected DM individuals. (**C**) Glutathione levels were significantly lower in non-DM and DM ICU COVID-19 patients than that in uninfected non-DM individuals. There were no significant differences in glutathione levels in non-DM and DM ICU COVID-19 patients compared to uninfected DM individuals. (**D**) Plasma of glutathione levels in non-DM and DM ICU COVID-19 patients that died had significantly lower than that in patients that survived. There was also a significant difference in MG levels of non-DM and DM ICU COVID-19 that died compared to uninfected DM individuals. There was not a significant difference in glutathione levels between non-DM and DM ICU COVID-19 patients that survived and uninfected DM individuals. (**E**) Glo1 levels in plasma of non-DM and DM ICU COVID-19 patients were not significantly different from that of uninfected non-DM or uninfected DM individuals. (**F**) Plasma of Glo1 levels in non-DM and DM ICU COVID-19 patients that died had significantly lower than that in non-DM and DM ICU COVID-19 patients that survived. There were also significantly lower in Glo-1 plasma levels of non-DM and DM ICU COVID-19 patients that died compared to uninfected DM individuals. Data shown in (**A**, **C**, **E**) are mean ± S.E.M from each of the n = 30 uninfected controls (26.6% females), n = 34 for non-DM (33.3% females) and n = 31 for DM (25.8% females). Data shown in (**B**, **D**, **F**) are mean ± S.E.M for each of the n = 30 in uninfected controls (26.6% females), n = 18 in non-DM survived (44.4% females), n = 16 in non-DM died (30.7% females), n = 14 DM in survived (28.6% females), and n = 17 in DM died (37.5% females) groups. Statistical significances are shown above data points on each graph.

**Figure 6:**
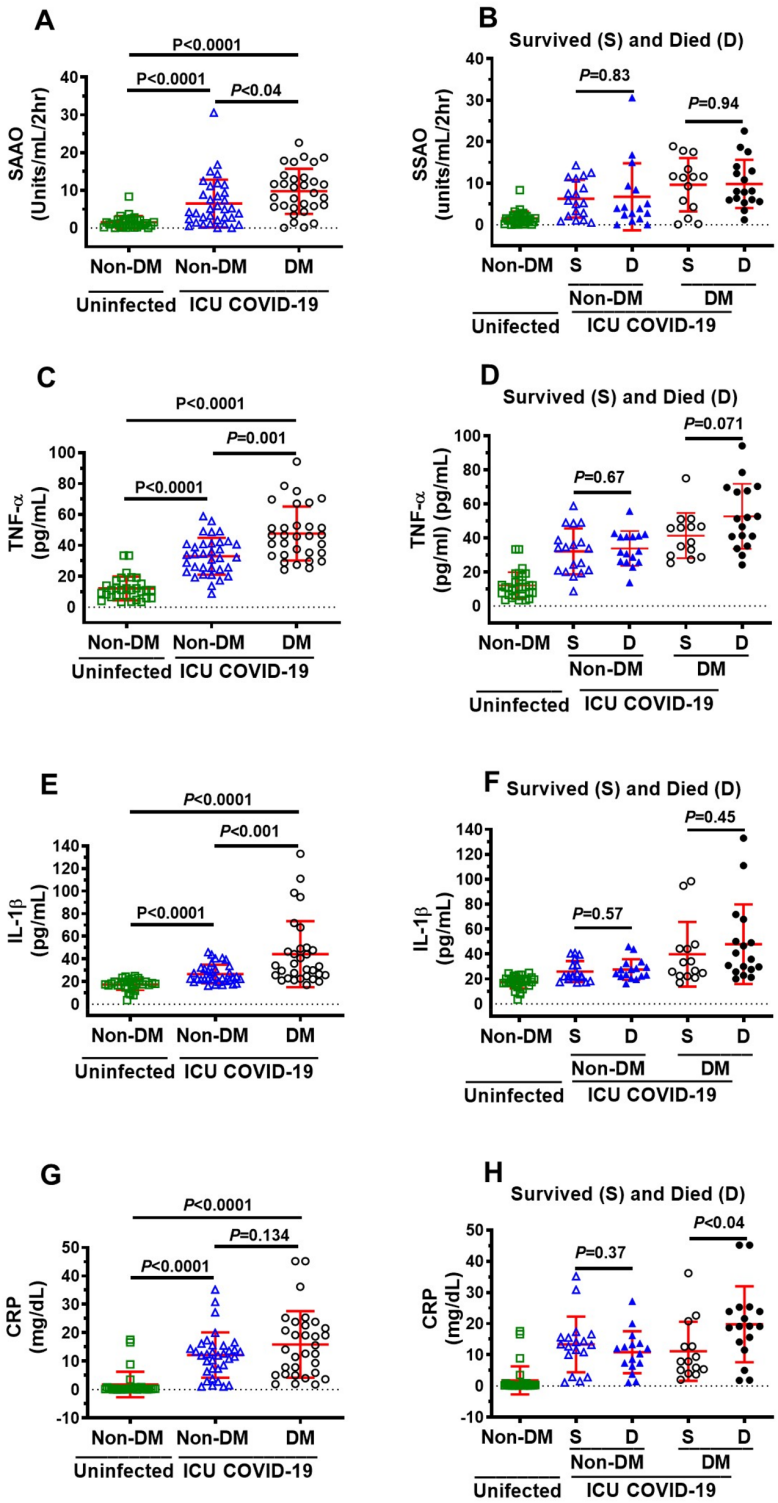
Plasma SSAO activities and levels of TNF-α, IL-1β and CRP in ICU COVID-19 patients without diabetes mellitus (non-DM) and with diabetes (DM) and separated into those that survived and those that died. (**A**) SSAO activities in plasma from non-DM and DM ICU COVID-19 patients were significantly higher than uninfected non-DM individuals. There was also a significant difference in plasma SSAO activities between non-DM and DM ICU COVID-19 patients. (**B**) No significant differences in SSAO activities in plasma from non-DM and DM ICU COVID-19 patients that died and survived. (**C**) TNF-α levels in plasma from non-DM and DM ICU COVID-19 patients were significantly higher than uninfected non-DM individuals. There was a significant difference in TNF-α in plasma from non-DM and DM ICU COVID-19 patients. (**D**) No significant differences in plasma levels of TNF-α in non-DM and DM ICU COVID-19 patients that died and survived. (**E**) IL-1β levels in plasma of non-DM and DM ICU COVID-19 patients were significantly higher than that in uninfected non-DM individuals. IL-1β levels in plasma from ICU COVID-19 with DM were also significantly higher than of ICU-COVID-19 patients without DM. (**F**) No significant difference in plasma levels of IL-1β in non-DM and DM ICU COVID-19 patients that died and survived. (**G**) Significantly higher CRP in plasma from non-DM and DM ICU COVID-19 patients compared to uninfected non-DM individuals. CRP in plasma from DM ICU COVID-19 was not significantly higher than non-DM ICU-COVID-19 patients. (**H**) Significant difference in plasma levels of CRP between DM ICU COVID-19 patients that died and survived. However, there was not significant differences in plasma levels of CRP in non-DM ICU COVID-19 patients between died and survived. Data shown in (**A**, **C**, **E**, **G**)are mean ± S.E.M from n = 30 in uninfected non-DM individuals (26.6% females), n = 34 in non-DM (33.3% females) and n = 31 in DM (25.8% females) group. Data shown in (**B**, **D**, **F**, **H**) are mean ± S.E.M from n = 30 in uninfected non-DM individuals (26.6% females), n = 18 in non-DM survived (44.4% females), n = 16 in non-DM died (30.7% females), n = 14 DM in survived (28.6% females), and n = 17 in DM died (37.5% females) groups. Statistical significances are shown above data points on each graph.

Studies have shown that patients with DM are at higher risk for severe COVID-19 outcomes compared with non-DM patients^11–13^. In this study, MG was 2.7-fold higher (1326 μg/ml HSA-MG in uninfected DM vs 495 μg/ml HSA-MG in uninfected control, *P* < 0.05), and glutathione was 2.3-fold (342 nmol/ml uninfected DM vs 777 nmol/ml in uninfected control, *P* < 0.05) lower in uninfected DM patients compared to uninfected non-DM patients (Fig. 5A,C). However, there was no significant difference in Glo1 levels between uninfected non-DM and uninfected DM patients (45 ng/ml uninfected DM vs 42 ng/ml in uninfected control) (Fig. 5E). MG was also significantly higher in COVID-19 DM ICU patients compared to uninfected DM patients (Fig. 5A). There were no significant differences in plasma levels of MG, glutathione, Glo1, and CRP between non-DM and DM patients upon ICU admittance (Figs. 5A,C,E, 6G). However, SSAO, TNF-α and IL-1β were significantly higher in plasma of DM ICU patients than non-DM ICU patients (Fig. 6A,C,E).

When non-DM and DM ICU patients were further subdivided into those that survived and died, plasma MG was significantly higher, and glutathione and Glo1 were significantly lower in non-DM and DM that died compared to ICU patients that survived (Fig. 5B,D,F). There were no differences between SSAO, TNF-α, and IL-1β levels between non-DM and DM patients that survived and died (Fig. 6B,D,F). CRP was higher in DM ICU patients that died than in DM patients that survived (Fig. 6H).

### Plasma levels of neutrophils, lymphocytes, monocytes, basophils and eosinophils in non-DM, uninfected DM and ICU COVID-19 patients

**Figure 7:**
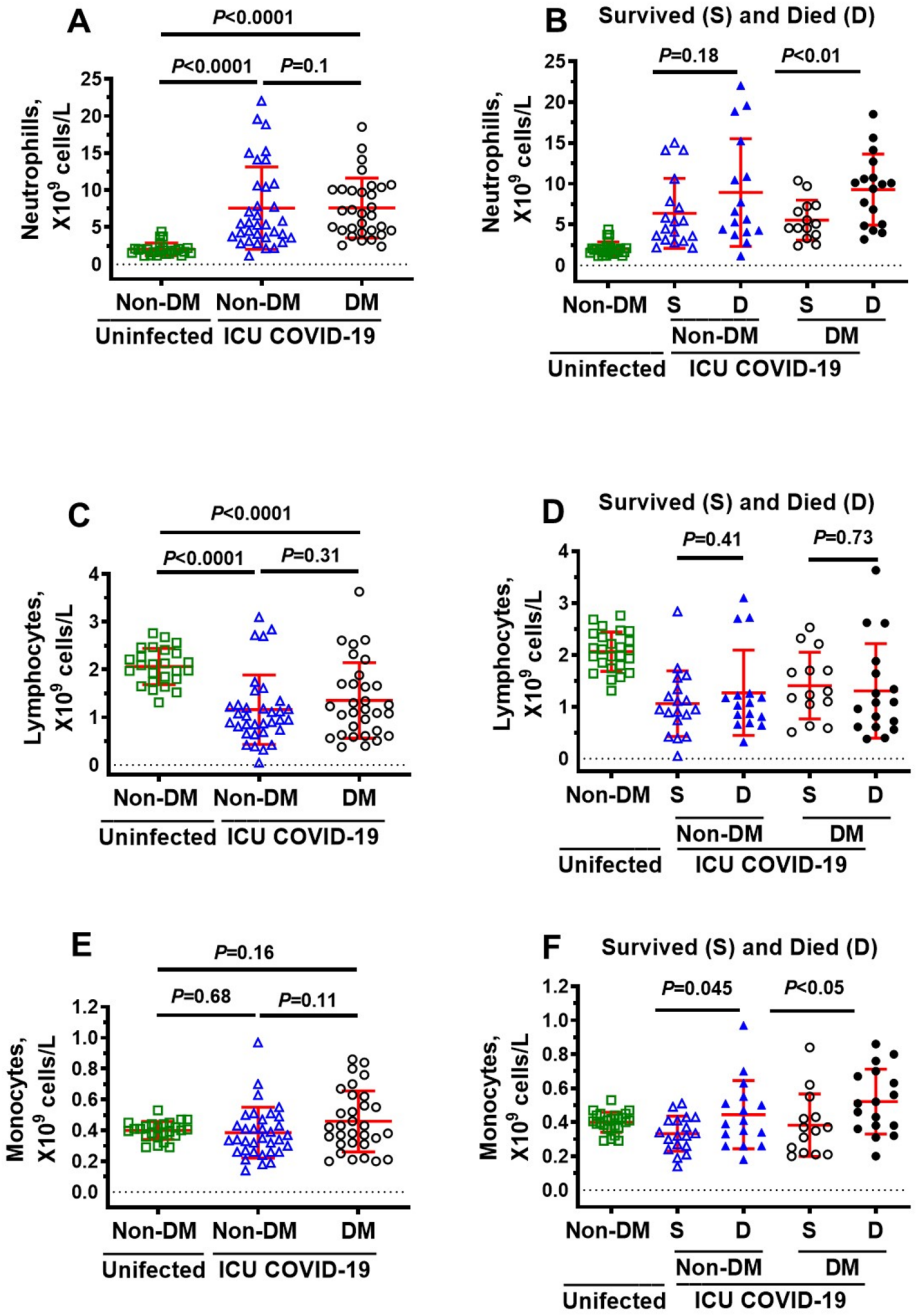
Blood levels of neutrophils, lymphocytes, and monocytes in ICU COVID-19 patients without diabetes mellitus (non-DM) and with diabetes (DM) and separated into those that survived and those that died. (**A**) Neutrophils levels in blood from non-DM and DM ICU COVID-19 patients were significantly higher than uninfected non-DM individuals. There was also a significant difference in blood neutrophils levels between non-DM and DM ICU COVID-19 patients. (**B**) No significant differences in neutrophils levels in blood from non-DM ICU COVID-19 patients that died and survived. However, there was a significant difference in blood neutrophils levels between DM ICU COVID-19 patients that survived and died. (**C**) Lymphocytes levels in blood from non-DM and DM ICU-COVID-19 patients were significantly higher than uninfected non-DM individuals. There was not a significant difference in lymphocytes levels in blood from non-DM and DM ICU COVID-19 patients. (**D**) No significant differences in blood levels of lymphocytes in non-DM and DM ICU COVID-19 patients that died and survived. (**E**) Monocytes levels in blood from non-DM and DM ICU COVID-19 patients were not significantly higher than uninfected non-DM individuals. Monocytes levels in blood from DM ICU COVID-19 were also not significantly higher than of non-DM ICU-COVID-19 patients. (**F**) Significant difference in blood levels of monocytes in non-DM and DM ICU COVID-19 patients that died and survived. Data shown in (**A**, **C**, **E**) are mean ± S.E.M from n = 30 in uninfected non-DM individuals (26.6% females), n = 34 in non-DM (33.3% females) and n = 31 in DM (25.8% females) group. Data shown in (**B**, **D**, **F**) are mean ± S.E.M from n = 30 in uninfected non-DM individuals (26.6% females), n = 18 in non-DM survived (44.4% females), n = 16 in non-DM died (30.7% females), n = 14 DM in survived (28.6% females), and n = 17 in DM died (37.5% females) groups. Statistical significances are shown above data points on each graph.

**Figure 8:**
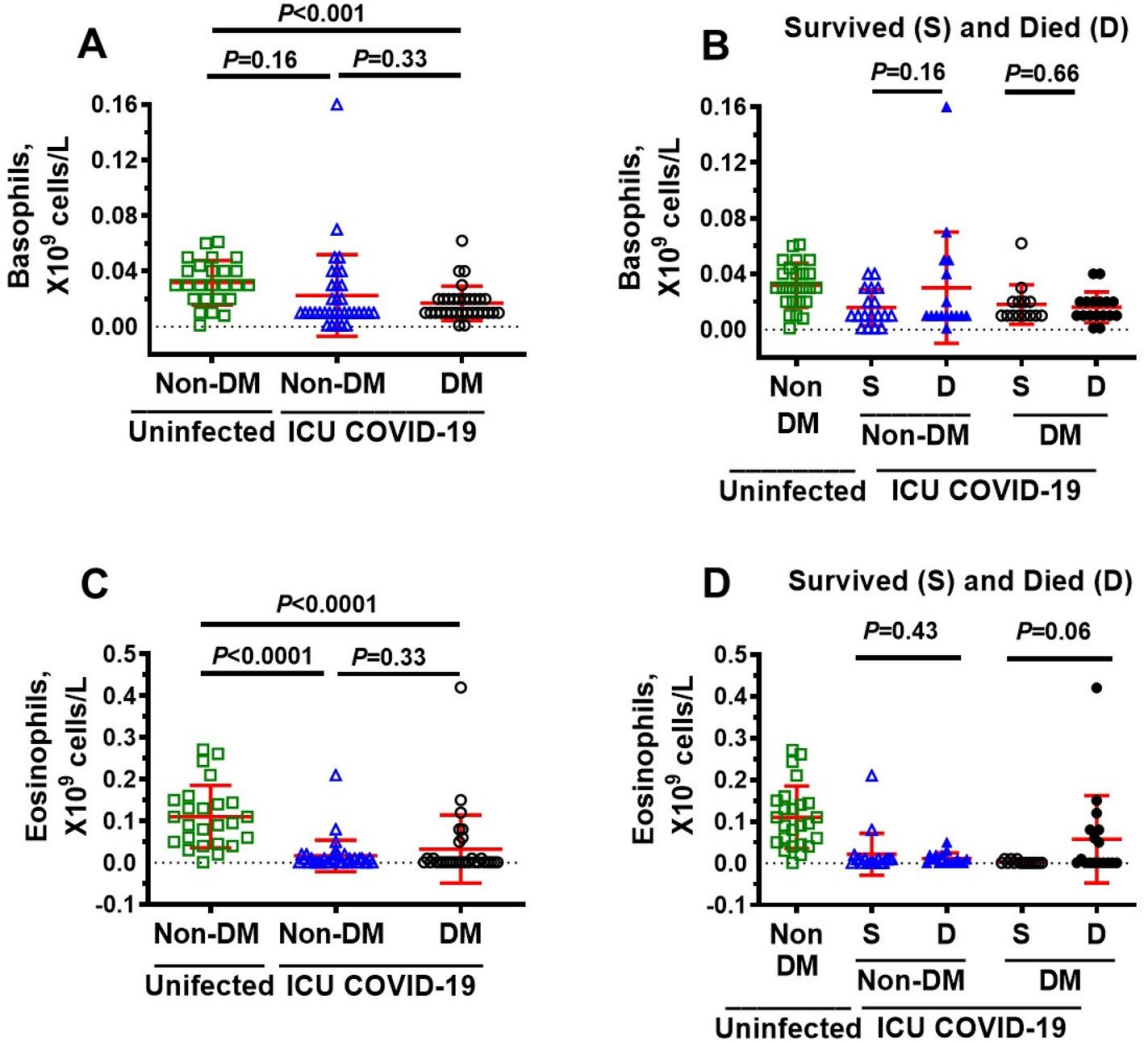
Blood levels of basophils and eosinophils in ICU COVID-19 patients without diabetes mellitus (non-DM) and with diabetes (DM) and separated into those that survived and those that died. (**A**) Basophils levels in blood from DM ICU-COVID-19 patients, but not non-DM ICU-COVID-19 were significantly higher than uninfected non-DM individuals. There was not a significant difference in blood basophils levels between non-DM ICU-COVID-19 and DM ICU-COVID-19 patients. (**B**) No significant differences in blood levels of basophils in non-DM and DM ICU COVID-19 patients that died and survived. (**C**) Eosinophils levels in blood from non-DM and DM ICU-COVID-19 patients were significantly higher than uninfected non-DM individuals. There was not a significant difference in eosinophils levels in blood from non-DM and DM ICU COVID-19 patients. (**D**) No significant differences in blood levels of eosinophils in non-DM and DM ICU COVID-19 patients that died and survived. Data shown in (**A**, **C**) are mean ± S.E.M from n = 30 in uninfected non-DM individuals (26.6% females), n = 34 in non-DM (33.3% females) and n = 31 in DM (25.8% females) group. Data shown in (**B**, **D**) are mean ± S.E.M from n = 30 in uninfected non-DM individuals (26.6% females), n = 18 in non-DM survived (44.4% females), n = 16 in non-DM died (30.7% females), n = 14 DM in survived (28.6% females), and n = 17 in DM died (37.5% females) groups. Statistical significances are shown above data points on each graph.

Next, we investigated whether neutrophils lymphocytes, monocytes basophils, and eosinophils levels are altered in non-DM and DM patients that survived and died. Compared to uninfected non-DM patients, the amount of neutrophil was higher, and the amounts of lymphocytes and eosinophils were lower in both non-DM and DM ICU patients, but not monocytes (Figs. 7A,C and 8C). Basophils levels were also significantly lower in DM but not in non-DM ICU patients compared to uninfected non-DM patients (Fig. 8A). In this study, there were also no significant differences in the amounts of neutrophils, lymphocytes, monocytes basophils and eosinophils between non-DM and DM COVID-19 patients (Figs. 7A,C,E, 8A,C). When non-DM and DM ICU patients were further subdivided into those that survived and died, we found higher levels of neutrophils in DM ICU patients that died compared to those that survived (Fig. 7B), and higher levels in monocytes in both non-DM and DM that died compared to non-DM and DM that survived (Fig. 7F). There were no significant differences in the number of lymphocytes and basophils in non-DM and DM COVID-19 ICU patients that died and survived (Figs. 7D, 8B) There was trend towards more eosinophils DM ICU patients that died compared to DM ICU patients that survived, but the data was not significant (*P* > 0.05, Fig. 8D).

### Mortality in ICU COVID-19 patients with low and high plasma MG

**Figure 9:**
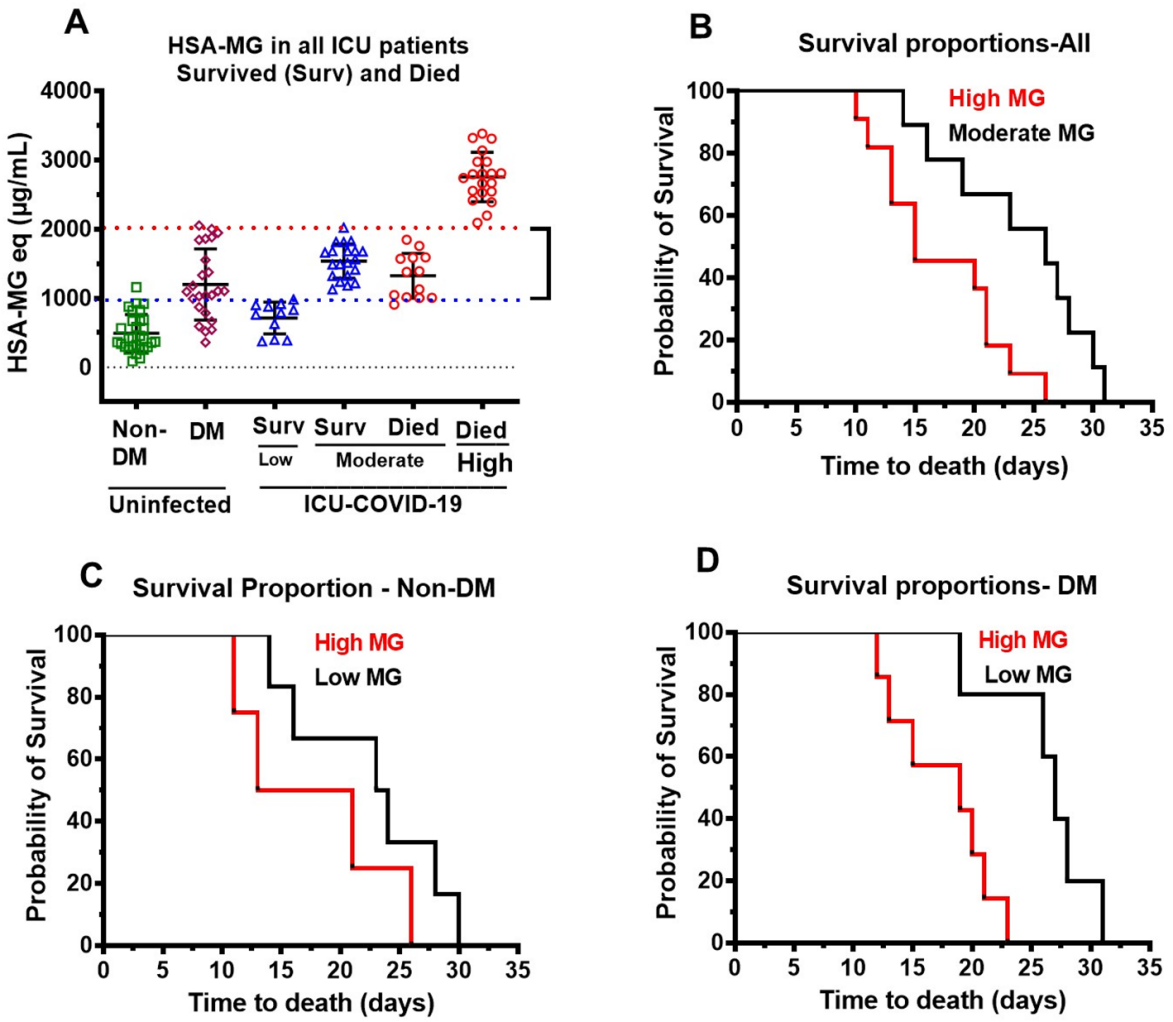
Kaplan–Meier curves for non-DM and DM ICU COVID-19 patients that died. (**A**) Low MG (< 2 times higher than uninfected non-DM), moderate MG (between 2 and 3 times higher than uninfected non-DM) and high MG (> 4times higher than uninfected non-DM) in ICU patients that survived and died along with MG in uninfected non-DM and DM individuals. Data shown are mean ± S.E.M for each of n = 30 uninfected controls (26.6% females), n = 11 survived for low, and n = 21 survived moderate. n = 13 died for moderate and n = 20 died, for high MG. (**B**) Kaplan–Meier survival curve for all ICU COVID-19 patients that died with moderate MG (n = 13) and high MG (n = 20). (**C**) Kaplan–Meier survival curve for non-DM ICU COVID-19 patients that died with moderate (n = 8) and high MG (n = 8). (**D**) Kaplan–Meier survival curve for DM ICU COVID-19 patients that died with moderate (n = 5) and high MG (n = 12).

Plasma MG levels were used to further separate ICU patients that survived into those with low MG (up to twofold higher than that in uninfected non-DM) and moderate MG (2–threefold higher than uninfected non-DM). MG level in uninfected non-DM 495 μg/ml HSA-MG, “□”) was used as the reference. For comparison, we also included MG levels in uninfected DM patients, “⋄” (Fig. 9A). All patients with low plasma MG upon admittance (11/65, 17%), were discharged from ICU. The median time to discharge was 7 days, range of 5–22 days. Patients (21/65, 32%) with moderate MG levels that survived had a mean time to discharge of 8.5 days, with a range of 3–32 days.

Patients that died were also divided into two group. The first group contained patients with MG less than fourfold than uninfected controls (moderate MG patients), and the second group contained patients with MG > fourfold than uninfected controls (high MG patients), Fig. 9A. In all patients with moderate MG that died, median time to death (13/65, 20%) was 25 days, range 14–31 days, and in the second group (20/65, 31%), the median time to death was 14 days with a range of 9–26 days. Figure 9B–D shows Kaplan–Meier curves for all patients, non-DM patients that died, and DM that died, respectively. These data show that the higher the plasma MG, the earlier the onset of death, regardless of whether the patient had DM or not.

A forward selection logistic regression model indicates evidence of a significant relationship between MG and COVID-19 patients (chi-square = 24.90, df = 1, P < 0.0001). Dead patients were found to have significantly higher MG (P < 0.0001) compared to their survived counterparts. The model correctly predicted 67% of death cases and explained 42% of the variability.

### Correlations between MG, glutathione, Glo1, SSAO, TNF-α, IL-1β, CRP, age and immune cells in ICU COVID-19 patients that died

**Figure 10:**
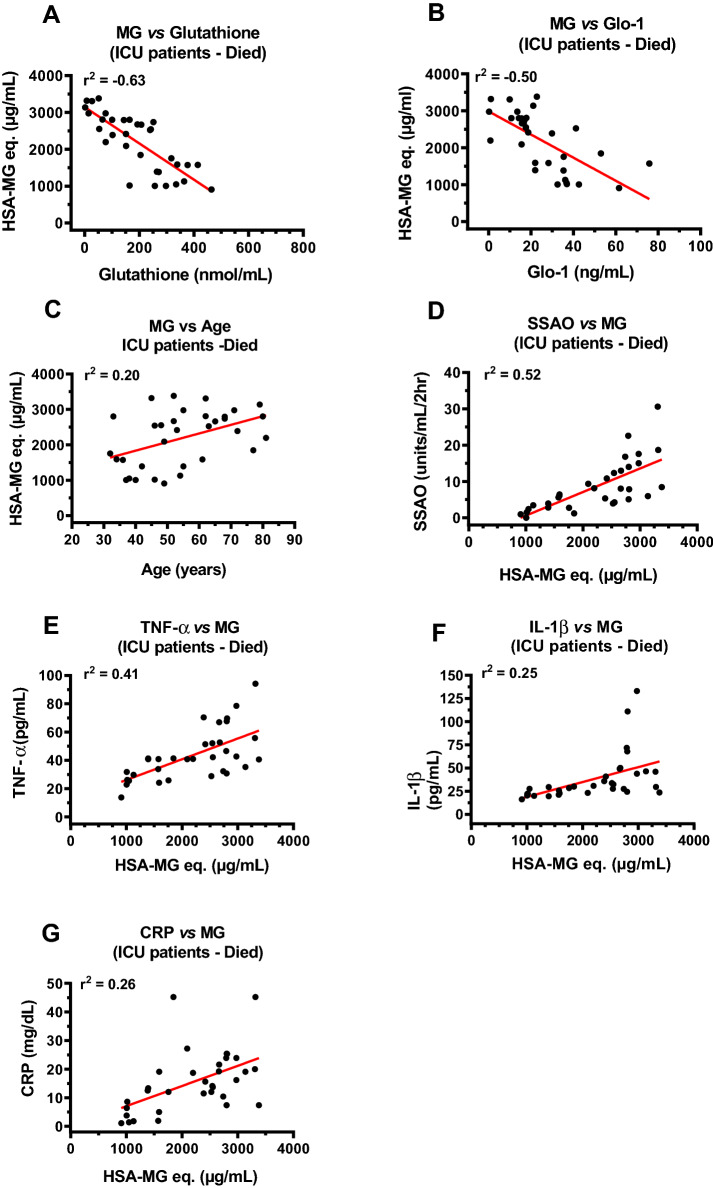
Correlations between plasma MG and glutathione, Glo1, SSAO, TNF-α, IL-1β and CRP in ICU patients that died. (**A**, **B**) Strong inverse correlation between MG and glutathione (r^2^ = − 0.63), and MG and Glo1 (r^2^ = − 0.50) in ICU COVID-19 patients that died. (**C**) Weak correlation between plasma MG and age (r^2^ = 0.20) in ICU COVID-19 patients that died. (**D**) Strong positive correlation (r^2^ = 0.52) between plasma MG and SSAO activity in ICU patients that died. (**E**) Moderate positive correlation (r^2^ = 0.41) between plasma MG and TNF-α in ICU COVID-19 patients that died. (**F**, **G**) Weak correlation between plasma MG and IL-1β (r^2^ = 0.25) and between MG and CRP (r^2^ = 0.26) in ICU COVID-19 patients that died. Data in graphs are for n = 33 patients.

**Figure 11:**
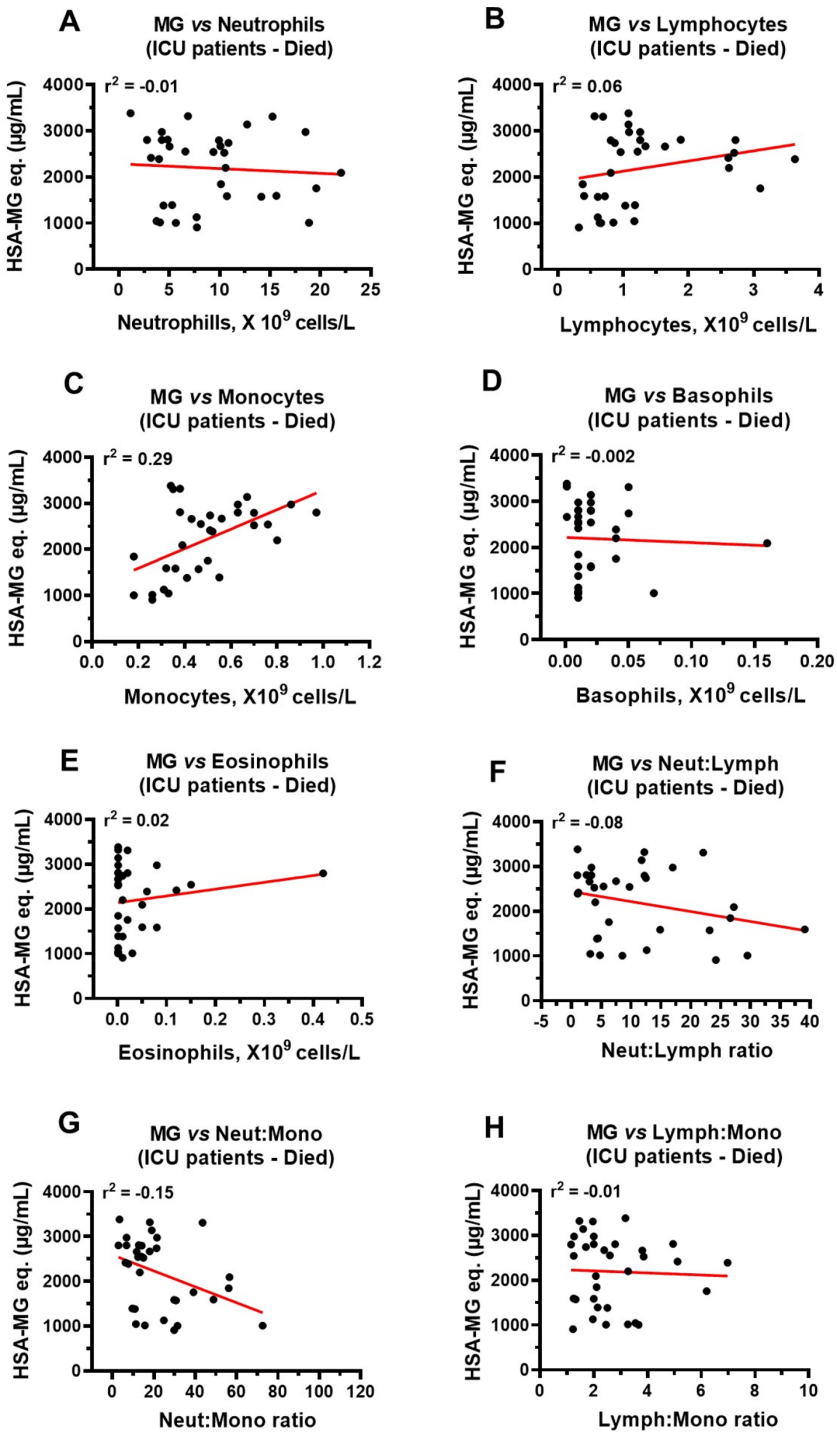
Correlations between plasma MG and neutrophils, lymphocytes, monocytes, basophils, and eosinophils in ICU patients that died. (**A**, **B**) No significant correlations between MG and neutrophils (r^2^ = − 0.01) and lymphocytes (r^2^ = 0.06) in ICU patients that died. (**C**) A significant correlation between plasma MG and monocytes (r^2^ = 0.29) in ICU COVID-19 patients that died. (**D**–**H**) No significant correlations between MG and basophils (r^2^ = − 0.002), eosinophils (r^2^ = 0.02), neutrophil:lymphocyte ratio (r^2^ = − 0.08) neutrophil:monocyte ratio (r^2^ = − 0.15) and lymphocytes:monocytes ratio (r^2^ = − 0.01), respectively in ICU COVID-19 patients that died. Data in graphs are for n = 33 patients.

Next, we investigated correlations between plasma levels of glutathione, Glo1, age and immune cells with MG levels to gain insights into reasons for MG accumulation, with glutathione, Glo1, age and immune cells as independent variables. Since MG induces inflammation^43,46^ we also investigated correlations between plasma MG and SSAO, TNF-α, IL-1β, and CRP levels with MG as the independent variable. In this study, strong inverse correlations were found between plasma MG and glutathione (r^2^ = − 0.63) and Glo1 (r^2^ = − 0.50), Fig. 10A,B. A weaker but significant positive correlation was also found between MG and age of patient (Fig. 10C). Strong positive correlation between plasma MG and SSAO (r^2^ = 0.52) and moderate correlation with TNF-α (r^2^ = 0.41) were found (Fig. 10D,E). Weaker but significant correlations were also observed with IL-1β (r^2^ = 0.25), and CRP (r^2^ = 0.26) (Fig. 10F,G). A strong positive correlation was also found between the number of monocytes and plasma MG levels in ICU patients that died (Fig. 11C). There were no significant correlations between MG and neutrophils, lymphocytes, basophils, eosinophils, neutrophil:lymphocyte and neutrophil:monocyte and lymphocyte:monocyte ratios (Fig. 11A,B,D–H).

## Discussion

About 10% of people infected with SARS-CoV-2 develop ARDS requiring intensive care hospitalization^3–5^. About 50% of COVID-19 ICU patients will also die^50^. To date, biomarkers to predict which ICU COVID-19 patients are at high risk of mortality are insufficient^4,15–19^. The principal finding of this cross-sectional study is that high plasma levels of the glycolysis byproduct MG upon admission into ICU with ARDS is a predictor of death in COVID-19 patients. This conclusion is based on our findings that mean plasma MG of COVID-19 patients admitted into the ICU that later died was 4.4-fold and 1.8-fold higher than that in uninfected non-DM controls and uninfected DM individuals, respectively. Similar levels of MG have been reported in plasma of uninfected controls^51^, Other have also reported similar fold increases in plasma MG in uninfected DM patients using other assay methods^52–54^. Mean plasma MG in COVID-19 patients on admission into ICU that survived, was 2.5-fold higher than that of uninfected non-DM controls, and not significantly different to that of uninfected DM patients.

Others have shown that glycolysis and the oxidative arm of the PPP are upregulated in SARS-CoV-2-infected cells to provide the substrates needed for replication^55,56^, and in immune cells to eliminate SARS-CoV-2 infection and repair any cellular damage^27–29^. As such, we concluded that MG synthesis is likely increasing in COVID-19 patients. In this study, we focused on whether MG degradation is being compromised in COVID-19 ICU patients. We found that plasma levels of glutathione, and Glo1 were significantly lower in ICU COVID-19 patients that died compared to ICU COVID-19 patients that survived. In an earlier report Horowitz et al.^57^ showed that oral and intravenous glutathione and the glutathione precursors (*N*-acetylcysteine) attenuated activation of NF-κB, cytokine storm syndrome and respiratory distress syndrome seen in COVID-19 patients with pneumonia. Thus, it is likely that intravenous glutathione and the glutathione precursors (N-acetylcysteine) administration were alleviating the oxidative stress and providing the glutathione needed for the formation of MG-glutathione hemiacetal. Glutathione is synthesized in two sequential reactions. In the first reaction, γ-glutamylcysteine ligase (GCL; EC 6.3.2.2) converts l-glutamate and l-cysteine into γ-glutamylcysteine, and in the second reaction, glutathione synthetase (GSS; EC 6.3.2.3) adds glycine to γ-glutamylcysteine to form glutathione^38,58,59^. In a recent study Moolamalla et al.^56^ found that the modifier and catalytic subunits genes of γ-glutamylcysteine ligase were downregulated in A549 (alveolar epithelial cells derived from lung adenocarcinoma), ACE2-induced A549, and normal human bronchial epithelial (NHBE) cells infected with SARS-CoV-2. These investigators also found downregulation of GSS gene in in A549, ACE2-induced A549, Calu3 (lung epithelial cells derived from lung adenocarcinoma) cells infected with SARS-CoV-2-infected and in lung biopsy from SARS-CoV-2-infected patients. Our findings are consistent with others showing that glutathione synthesis is being compromised in COVID-19 ICU patients. The increase in oxidative stress induced by SARS-CoV-2 infection may also be lowering the amount of reduced glutathione in host cells/tissues.

Additionally, we also found a reduction of plasma levels of Glo1 protein in COVID-19 patients that died. Moolamalla et al.^56^ reported down regulation of Glo1 gene in ACE2 transduced A549 and Calu3 cells infected with SARS-CoV-2. To date, the underlying cause(s) for the reduction plasma Glo1 levels in plasma of COVID-19 patients that died remain poorly defined. What we know is that the promoter region of human *GLO1* has a functionally operative antioxidant response element (ARE)^39–41^. Under non-stress conditions, the antioxidant transcription factor nuclear factor erythroid 2-related factor 2 (Nrf2) binds to the ARE region of *GLO1* to induce its expression. NF-κB antagonizes the binding of Nrf2 to ARE to inhibit Glo1 expression. Since activation of NF-κB is upregulated in COVID-19 patients^60^, the increase in activated NF-κB could also account in part for the reduction plasma Glo1. HIF-1α also binds to the ARE of *GLO1* to suppress Glo1 expression^39,41^. HIF-1α is one of the two subunits of the heterodimeric transcription factor that regulates cellular and systemic adaptive responses to low oxygen (hypoxia)^24,28,30,33^. HIF-α stabilization can also occur under normoxia during immunity and inflammation via upregulation of PI3K, AKT, mTOR, and STAT3 pathways in polarized M1 macrophages^30^. When oxygen delivery is compromised as is the case with respiratory distress syndrome and ischemia in COVID-19, HIF-1α escapes degradation, allowing it to migrate to the nucleus and induce transcription of HIF-1α target genes, including those involved in glycolysis and erythropoiesis^33,61,62^. The increase in glycolysis and suppression of Glo1 expression by HIF-1α inadvertently leads to an increase in MG. To the best of our knowledge, there is no published literature on linking HIF-1α upregulation and elevation in MG in COVID-19 patients.

In ICU patients that died, we also found blood neutrophils were 450% (*P* < 0.0001) and monocytes were 20% (*P* < 0.05) higher than that in non-infected controls. Lymphocytes and eosinophils were also 37% and 68% lower than that in non-infected controls, respectively. Basophil levels were not significantly different between uninfected controls and ICU COVID-19 patients. Other have reported neutrophilia, lymphopenia and monocytosis in COVID-19 patients were associated with poor outcomes^63^. While it is clear that high levels of neutrophils and monocytes arise from the body’s response to eliminate SARS-CoV-2^31,32,60,64^, specific mechanisms by which neutrophilia and monocytosis contribute to poor outcomes in COVID-19 patients remain poorly understood. We posit that neutrophilia and monocytosis could be contributing to poorer outcomes in COVID-19 patients in part by increasing production of the cytotoxic glycolysis metabolite MG.

In this study, all ICU COVID-19 patients had significantly higher plasma levels of the inflammation markers originating from activation of several pathways; SSAO from increased expression of the inflammation-induced protein vascular adhesion protein-1^48^, TNF-α from activation of nuclear factor kappa-light-chain-enhancer of activated B cells (NF–κB)^65^, and IL-1β from activation of the inflammasome^66–68^ compared to uninfected non-DM individuals. However, when ICU COVID-19 patients were subdivided into those that survived and died, there were no significant differences between SSAO, TNF-α, and IL-1β, suggesting that these inflammation biomarkers are not predictive of death.

Individuals with DM are at increased risk of severe respiratory and adverse outcomes including death following SARS-CoV-2 infection compared to non-DM patients^6,10–12^. This prompted us to separate our ICU cohorts further into non-DM and DM and with subdivision into those that survived and those that died. In this study, the amount of MG in plasma of ICU COVID-19 patients without DM were not significantly different from that in uninfected DM patients. However, ICU COVID-19 patients with DM had significantly higher levels of plasma MG than uninfected DM patients. MG levels in ICU COVID-19 with and without DM patients that died were 70% and 68% higher than that in non-DM and DM that survived, respectively. As in this study, other have also reported decreased glutathione and Glo1 level in DM patients compared to non-DM patients^69,70^. However, in this study, glutathione and Glo1 levels in ICU COVID-19 patients with and without DM that died were significantly lower than that in ICU COVID-19 patients with and without DM that survived and uninfected DM, indicating that the degradation of MG is being compromised in non-DM and DM patients that died.

To gain further insight into the relationship between plasma MG levels and death, the logistic regression analysis revealed evidence of a significant relationship between MG and COVID-19 patients that died. Our model also correctly predicted 67% of death cases in ICU COVID-19 patients and explained 42% of the death variability. Correlational studies were also conducted to investigate the relationships between plasma MG as the dependent variable. We found strong inverse correlations between MG and glutathione, Glo1, and age. As an independent factor, MG also positively correlated with SSAO and TNF-α. These data suggest MG elevation is arising from both an increase in synthesis via glycolysis and from impaired degradation due to reduction in glutathione and Glo1. To the best of our knowledge, these data are the first to show that elevated plasma MG in ICU COVID-19 patients upon admission is predictive of death.

This study is not without limitations. ELISA assays were used for measuring MG (HSA-MG), Glo1, TNF-α, and IL-1β. Although the protocols for these assays were provided by the manufacturer and followed as per instruction with appropriate controls, additional work studies are needed using other methodologies, including mass spectrometry, Western blot assays and quantitative polymerase chain reactions (q-PCR) for measurements of MG, Glo1, TNF-α, and IL-1β.

**Figure 12:**
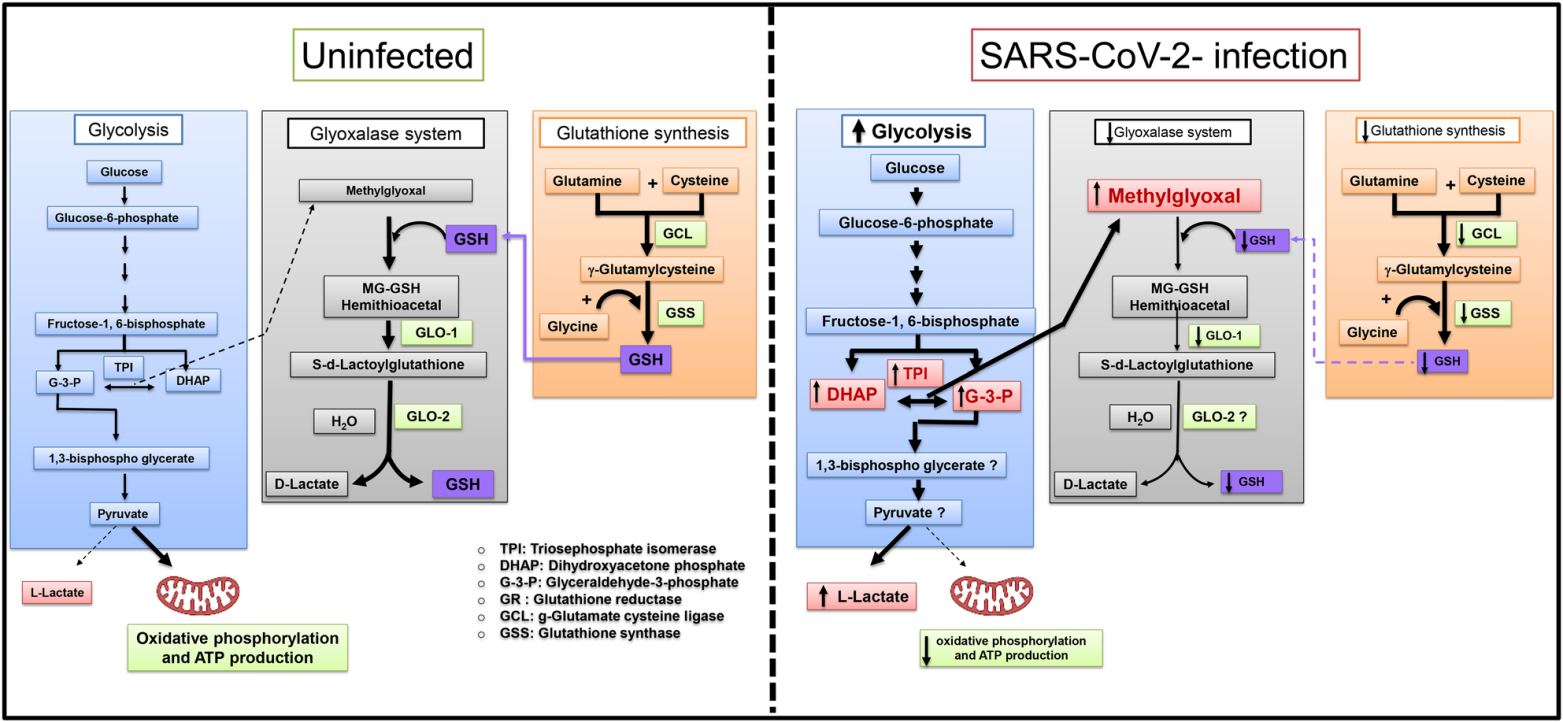
Overview of methylglyoxal (MG)formation via glycolysis and the degradation of the methylglyoxal-glutathione hemiacetal in uninfected and SARS-CoV-2 infected individuals. MG is formed from the interconversion of the dihydroxyacetone phosphate (DHAP) and glyceraldehyde-3-phosphate (G3P) via triosephosphate isomerase that generates MG. MG is detoxified by the dual-enzyme glyoxalase system. In the first step, the rate-limiting glyoxalase 1 (Glo1) converts the hemithioacetal formed between MG and reduced glutathione (MG-GSH) to the thioester S-d-lactoylglutathione. In the second step glyoxalase 2 (Glo2) enzyme catalyzes the hydrolysis of S-d-lactoylglutathione to form d-lactate. During this reaction, GSH is recycled. Following SARS-CoV-2 infection, glycolysis is upregulated in the infected cells and host immune cells. At the same time, Glo1 and GSH are down regulated, resulting in cytotoxic levels of MG.

In summary, the present study shows for the first time that elevation in plasma levels of the cytotoxic glycolysis metabolite MG can be used as a novel independent biomarker that predicts mortality in ICU COVID-19 patients. This elevation in MG is arising from increased glycolysis in SARS-CoV-2 infected and immune cells and from impairment in MG degradation due to down regulation of Glo1 and glutathione. Our working hypothesis is detailed in Fig. 12. Since elevated MG is cytotoxic to cells, we posit that therapeutic strategies to lower MG levels may be useful in reducing adverse clinical outcomes in SARS-CoV-2 infection. These new data suggest that post COVID syndrome may be due in part to vascular and tissue damage initiated by elevated MG levels.

## Materials and methods

### Study participants

This study was approved by the ethical committee of Imam Abdulrahman Bin Faisal University, Al Kubar (IRB # 2020-05-184), and of Qatif Central Hospital (QCH-SREC0229/2020). COVID-19 patients admitted to the ICU in Qatif Central Hospital between October 2020 to Feb 2021 were recruited into this study after informed consent was taken. All ICU patients had respiratory rate ≥ 30 beats/min; blood oxygen saturation ≤ 93% at rest; arterial oxygen partial pressure (PaO_2_)/oxygen concentration (FiO_2_) ratio < 300; lung infiltrates > 50% of the lung field within 24–48 h. SARS-CoV-2 infection was confirmed by two sequential real-time reverse transcriptase polymerase chain reaction assays (Abbott Molecular Real Time SARS-CoV-2 assay, Abbott Molecular, Des Plaines, IL, USA) from nasopharyngeal, oropharyngeal and bronchoalveolar lavage fluid swab specimens. Age, medical history, and prior medications were obtained from medical records. The attending physicians reported the outcome of the ICU patients as either dead or discharge. Uninfected volunteers (non-diabetic and diabetic) were recruited from Al-Ahsa and Qatif areas to serve as controls. All assays were performed in accordance with guidelines for handling and assaying blood samples from COVID-19 patients by Imam Abdulrahman Bin Faisal University.

### Blood sampling

Two blood samples were obtained by a registered nurse from COVID-19 infected patients the morning after admittance (8:00 and 9:30 a.m.) and from uninfected individuals upon arrival (also between 8:00 and 9:30 a.m.) in EDTA anticoagulant tubes. One sample of blood from each individual was sent to the hospital laboratory for measurements of glucose, hemoglobin, hematocrit, white blood cells, red blood cells, neutrophils, lymphocytes, monocytes, basophils, eosinophils, platelets sodium, potassium, chloride, calcium, magnesium, albumin, urea, creatinine, total bilirubin, alanine aminotransferase, aspartate aminotransferase, creatine kinase, creatinine kinase-myocardial band, prothrombin time, activated partial thromboplastin time, international normalized ratio, lactate, ferritin, C-reactive protein, total cholesterol, triglyceride, high density lipoprotein, and low density lipoprotein. The second blood sample from each patient was centrifuged at 3500 rpm for 5 min at 4 °C in a refrigerated centrifuge and plasma was collected, aliquoted and stored at − 80 °C for measurements of the following below biomarkers.

### Methylglyoxal (HSA-MG equivalent) in plasma

Levels of human serum albumin-methylglyoxal adduct were measured in plasma samples using the competitive methylglyoxal (MG) ELISA kit (Hycult Biotech, Inc, Wayne, PA, USA, catalog # HIT503) based on the inhibition principle. In brief, plasma samples were thawed at room temperature and vortexed 30 s. Different standard concentrations and diluted plasma (1:5) were preincubated with labeled anti-MG trace antibody in U-shaped microtiter plate at room temperature. After 1 h, 100 μL of standard or samples mixed with tracer from the U-shaped plate were transferred in duplicate into appropriate wells in the coated microtiter plate coated with MG-adduct and incubated for 1 h at room temperature. After three washing, diluted streptavidin- Horseradish peroxidase (HRP) was added to each well and plate was incubated for 60 min at room temperature. Plate was washed three time and chromogenic substrate 3,3,5,5-tetramethylbenzidine (TMB) which catalyzes by HRP to generate a blue color was added. After 30 min, a 100 μL of acidic stop solution was added into each well and the absorbance at 450 nm was recorded using a using a Biotek Synergy Neo2 HTS Multi-Mode Microplate Reader (Männedorf, Switzerland). A standard curve was generated, and MG-adduct concentrations were calculated.

### Glyoxlase-1 (Glo1) in plasma

Glyoxalase-I (Glo1) levels in plasma from uninfected and COVID-19 were detected using commercial ELISA kits according to the manufacturer’s protocols (MyBioSource, Inc., San Diego, CA, USA, catalog # MBS2021816). Briefly, 100-μL of different concentration standards or diluted plasma samples (1:10) were added to the wells in duplicate, and the plate was incubated for 60 min at 37 °C. After this, 100-μL of detection reagent A was added to each well, and the plate was incubated for 60 min at 37 °C. Thereafter, the plate was washed three times and a 100-μL of detection reagent B working solution was added to each well, and the plate was incubated for 30 min at 37 °C. After 5 washes, 90-μL of TMB substrate solution was added into each well, incubated for 20 min at 37 °C. Finally, 50-μl of stop solution was loaded into each well and the absorbance was measured at 450 nm using the Biotek Synergy Neo2 HTS Multi-Mode Microplate Reader. The intensity of the color product was directly proportional to concentration of Glo-1 in plasma.

### Glutathione in plasma

The total glutathione concentration in plasma was determined using the glutathione assay Kit (Sigma-Aldrich, Inc., St Louis, MO, USA, catalog #. CS0260). First, the plasma samples were deproteinized with equal volume of the 5% 5-sulfosalicylic acid solution, vortexed vigorously for about 30 s, incubated for 10 min at 4 °C, centrifuged at 10,000 × *g* for 10 min at 4 °C and supernatants were collected. A glutathione standard curve from 1.56 to 50 nmol was prepared. To each well of a 96-well plate, 150 μL of working mixture and a 10 μL of standard or 100 μL of plasma were added in duplicate. Plate was incubated for 5 min at room temperature and then 50 μL of nicotinamide adenine dinucleotide phosphate (NADPH) solution (0.16 mg/mL) was added. The absorbance at 412 nm was recorded using a microplate reader. A standard curve was generated by linear regression and glutathione concentrations were calculated.

### Tumor necrosis factor alpha (TNF-α) in plasma

Tumor necrosis factor alpha (TNF-α) level in plasma was measured using an ELISA kit (Abcam Inc, Cambridge, MA, USA, catalog # Ab181421,) according to the manufacturer’s instruction. Briefly, a 50-μL of standard or 50-μL of undiluted tested samples were added to the wells in duplicate and then a 50-μL of antibody cocktail (a mixture of capture and detector antibody) was added to each well. The plate was incubated for 1 h at room temperature on a plate shaker stetted to 400 rpm. After 3 washing, 100-μL of TMB substrate was loaded and incubated for 10 min in the dark on a plate shaker stetted to 400 rpm. Finally, 100-μL of stop solution was added to each well and the optical density (OD) was recorded at 450 nm using a Biotek ELX 800 microplate reader.

### Interleukin-1 beta (IL-1β) levels in plasma

Plasma level of human interleukin-1 beta (IL-1β) was measured according to the manufacturer’s protocols (Abcam Inc, Cambridge, MA, USA, catalog # Ab 46052). A 100-μL of standard, control and tested samples in duplicate were added to the wells. Biotinylated anti-IL-1β was then added, and the plate was incubated for 3 h at room temperature. After 3 washings, 100-μL of streptavidin- HRP solution was added into all wells and incubated for 30 min. Next, plate was washed 3 times and each well was incubated with 100 μL of TMB in the dark for 15 min at room temperature followed by 100-μL of acidic stop solution. The absorbance at 450 nm was recorded using the microplate reader.

### Semicarbazide-sensitive amine oxidase (SSAO) activity in plasma

The plasma levels of semicarbazide-sensitive amine oxidase (SSAO) were determined using the Fluoro-SSAO assay Kit (Cell Technology, Inc. Hayward, CA USA, catalog # SSAO100-3). In this experiment, all plasma tested samples were diluted in ratio of 1:5 by reaction buffer. Since benzylamine served as a substrate for both SSAO and monoamine oxidase B, pargyline, a monoamine oxidase B inhibitor, was then added to a final concentration of 0.5 mM to each sample and incubated for 30 min at 37 °C. In a black 96-well plate, 100μL of standard or sample were added to each individual wells. Thereafter, 100μL of the reaction cocktail, a mixture of detection reagent, HRP and benzylamine were added to each well and incubated at 37 °C for 2 h. After 2 h, the plate was read with excitation at 530–570 nm and emission at 590 nm using Biotek Synergy Neo2 HTS Multi-Mode Microplate Reader.

### Statistical analyses

Data were analyzed using GraphPad Prism 7.0 software (La Jolla, CA) and Statistical Package for Social Sciences (SPSS) version 26.0. Armonk, NY: IBM Corp, and presented in text as mean $\pm$ standard error of the mean (SEM). T-test for independent samples or one-way ANOVA with Brown-Forsythe and Bartlett tests were used for continuous data. Logistic regression analysis was also used to explore the influence of the examined variables on death/survival. Significant differences were considered at *P* < 0.05.

## Data Availability

Data are available from the corresponding author upon reasonable request. FAA and KRB is the guarantors of this work, has full access to all the data in the study and takes responsibility for the integrity of the data and the accuracy of the data analyses.
